# PD-1 and PD-L1 Expression in Endometrial Cancer: A Systematic Review of the Literature

**DOI:** 10.3390/jcm14020401

**Published:** 2025-01-10

**Authors:** Orazio De Tommasi, Matteo Marchetti, Marta Tripepi, Sofia Bigardi, Giosuè Giordano Incognito, Valentina Tuninetti, Emma Facchetti, Giulia Tasca, Marco Noventa, Carlo Saccardi, Roberto Tozzi, Giulia Spagnol

**Affiliations:** 1 Unit of Gynecology and Obstetrics, Department of Women and Children’s Health, University of Padua, 35122 Padua, Italy; matteo.marchetti@unipd.it (M.M.); marta.tripepi@studenti.unipd.it (M.T.); sofia.bigardi@studenti.unipd.it (S.B.); emma.facchetti@studenti.unipd.it (E.F.); carlo.saccardi@unipd.it (C.S.); roberto.tozzi@unipd.it (R.T.); giulia.spagnol@unipd.it (G.S.); 2 Department of General Surgery and Medical Surgical Specialties, University of Catania, 95123 Catania, Italy; giordanoincognito@gmail.com; 3 Department of Oncology, Ordine Mauriziano Hospital, University of Turin, 10124 Turin, Italy; valentina.tuninetti@unito.it; 4 Istituto Oncologico Veneto IOV-IRCCS, 35128 Padova, Italy; giulia.tasca@iov.veneto.it

**Keywords:** endometrial cancer, microsatellite instability, immunotherapy, PD-1, PD-L1, mismatch repair

## Abstract

**Background/Objectives:** Cancer immunotherapy through the use of PD-1/PD-L1 inhibitors have shown significant promise in endometrial carcinoma (EC), particularly in tumors with microsatellite instability (MSI) or mismatch repair deficiency (dMMR), present in approximately 30% of cases. This review evaluated PD-L1 and PD-1 expression as potential biomarkers for immunotherapy response in EC, focusing on their relationship with MSI status. **Methods:** A systematic review, adhering to PRISMA guidelines, analyzed studies from MEDLINE and Embase until February 2023 on PD-1/PD-L1 expression in EC stratified by MSI status, including diverse study designs but excluding conference abstracts, with independent screening, data extraction, and additional reference checks to ensure comprehensive coverage. **Results:** A systematic analysis of 10 studies found that PD-L1 expression was more frequently expressed in MSI tumors (49%) compared to microsatellite-stable tumors (MSS) (33.5%), while PD-1 was expressed in 58% of MSI cases and 48% of MSS cases. Despite these findings, the prognostic value of PD-L1/PD-1 remains uncertain, with conflicting results regarding their association with survival outcomes. PD-L1 expression varied across molecular subtypes, being highest in POLE-mutated tumors (76.56%) and serous carcinomas (73%). Differences in PD-L1 expression between primary and metastatic sites were also noted, complicating its use as a biomarker. **Conclusions:** The assessment of PD-L1 expression in EC could represent a valuable option for selecting patients who may benefit from immune checkpoint inhibitors (ICI), including those in the MSS cohort, thereby ensuring a more tailored and personalized treatment strategy.

## 1. Introduction

**Figure 1:**
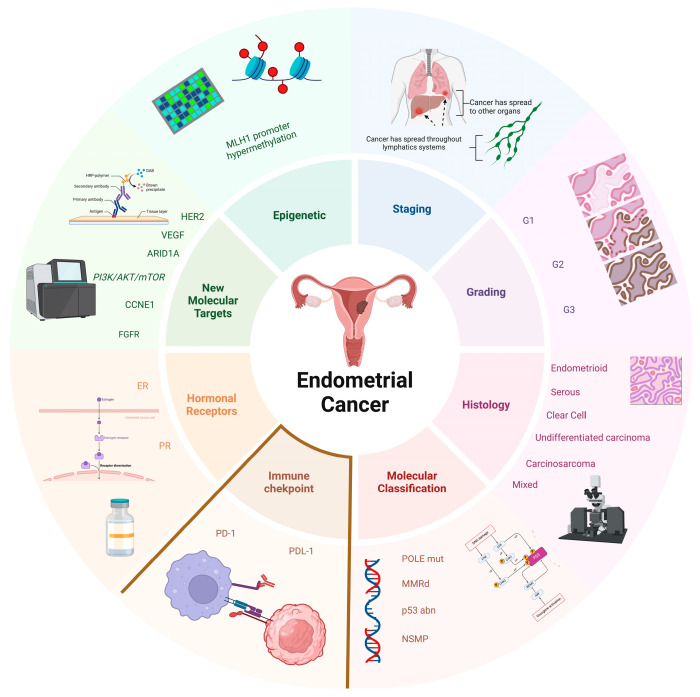
This image highlights the array of factors guiding the treatment of endometrial carcinoma. In addition to traditional parameters such as staging, grading, and histology, the presence of genetic or epigenetic alterations has become indispensable for determining the risk classification of endometrial cancers. Furthermore, the potential investigation of the expression of certain molecular markers (e.g., HER2), immune checkpoints such as PD-L1 and PD-1, as well as hormone receptors (ER, PR), could serve as a valuable tool for clinicians to deliver increasingly tailored therapies to patients. *POLE mut: Polymerase epsilon-mutated; dMMR: Mismatch Repair Deficient; p53 abn: Abnormal p53 expression; NSMP: No Specific Molecular Profile; PD-1: Programmed Death-1; PD-L1: Programmed Death-Ligand 1; ER: Estrogen Receptor; PR: Progesterone Receptor.*

Endometrial cancer (EC) is the most prevalent gynecologic malignancy in industrialized countries, with both incidence and mortality rates increasing over the past few decades [1]. EC is typically diagnosed at an early stage, where the prognosis is favorable, and surgery serves as the primary treatment modality. However, for advanced or recurrent EC, systemic chemotherapy with carboplatin and taxane has long been the standard of care [2]. In recent years, novel advancements have significantly enriched the diagnostic and therapeutic landscape of EC, revolutionizing its clinical management (Figure 1).

Programmed death-ligand 1, also known as PD-L1 (CD274), is a transmembrane protein primarily expressed in various tumor cells, tumor-infiltrating cells, and antigen-presenting cells (APCs) [3]. Programmed death 1 (PD-1) is a key immunosuppressive target primarily found in macrophages, B lymphocytes, dendritic cells (DCs), monocytes, tumor-specific activated T cells, myeloid cells, and natural killer (NK) cells [4]. Tumor cells utilize immune evasion strategies by upregulating PD-1 and PD-L1, which suppress T cell activity. The interaction between PD-1, expressed on T cells, and PD-L1, present on tumor cells, prevents T cell activation, thereby enabling tumors to evade immune destruction and continue proliferating [5]. This understanding has led to the development of immune checkpoint inhibitors (ICIs), which disrupt these interactions, reactivating T cells and restoring the immune system’s ability to target and eliminate tumor cells. Immuno-oncology with PD-1/PD-L1 targeting agents has been extensively studied across various tumor types and has become standard clinical practice in many of them [6]. More recently, efficacy data have also emerged for EC, demonstrating its potential as a treatment option for patients with advanced disease [7,8].

The TCGA project has significantly advanced the understanding of EC’s molecular landscape. By analyzing genomic, transcriptomic, and proteomic data from over 370 EC samples, TCGA categorized the disease into four molecular subtypes: POLE-ultramutated, microsatellite instability-high (MSI-H), copy-number low, and copy-number high. This classification is defined by distinct genetic and molecular features that influence prognosis and treatment responses. In detail, POLE-ultramutated tumors, marked by a high mutation rate due to POLE gene mutations, exhibit strong immunogenicity and favorable outcomes. MSI-H tumors, resulting from mismatch repair deficiencies, show high mutational loads and are responsive to immunotherapy. Copy-number low tumors, often hormone receptor-positive, tend to have a more indolent course, while copy-number high tumors, associated with serous histology and TP53 mutations, are linked to poor prognosis [9].

Building on these insights, the ProMisE classification system has offered a clinically applicable framework to align with TCGA subtypes using surrogate markers. It has stratified ECs into four molecular groups: mismatch repair-deficient (dMMR), POLE-ultramutated (POLEmut), p53-abnormal (p53abn), and no specific molecular profile (NSMP) [10].

With up to 30% classified as mismatch-repair-deficient/high microsatellite instability (dMMR/MSI-H), EC has the highest rate of dMMR status among human cancers [11]. Mismatch repair deficiency occurs either as an inherited mutation (in patients with Lynch syndrome) in at least one of the mismatch repairs (MMR) genes, MLH1, PMS2, MSH2, and MSH6, or as sporadic methylation of the MLH1 promoter. Defects in the MMR genes result in the inability to correct DNA replication errors, leading to dMMR/MSI-H characterized by a high somatic mutation rate [12]. It has also been demonstrated that dMMR/MSI-H EC is associated with a large number of tumor-infiltrating lymphocytes (TILs) compared to mismatch-repair-proficient/microsatellite-stable (pMMR/MSS) EC [13]. Given the increasing focus on the immune environment in EC, several clinical trials tested ICIs in this setting, especially for patients with metastatic or recurrent disease [14]. While clinical research initially began to test immunotherapy in EC regardless of MMR status, patients with dMMR/MSI-H tumors appeared to have better responses to ICIs [15]. Currently, MMR deficiency serves as the primary predictive biomarker for selecting EC patients who may benefit from immunotherapy.

However, the expression profiles of PD-L1 and PD-1, particularly in primary tumors and metastatic lesions, remain insufficiently investigated in EC. The objective of this review is to analyze PD-L1 and PD-1 expression in ECs across different molecular and histological subgroups, with a specific focus on their relationship to MMR status.

## 2. Materials and Methods

A systematic review of the literature was performed according to the Preferred Reporting Items for Systematic Reviews and Meta-analyses (PRISMA) guidelines [16]. We searched the electronic databases MEDLINE and Embase from inception until February 2023, using the MeSH terms (Medical subject headings) in MEDLINE and the Emtree-terms (Embase subject headings) in Embase, along with synonyms for both databases. The literature search was performed by a professional librarian of the Padua University using various combinations of the following terms: (“endometrial cancer”, OR “endometrial neoplasm”, OR “endometrial tumor”, OR “endometrial carcinoma”) AND (“PD-L1” OR “PD-1”) AND (“MSI” OR “mismatch repair-deficient” OR “Microsatellite instability” OR “dMMR”) AND (“MSS” OR “microsatellite stability”). We considered all studies evaluating the expression of PD-L1 or PD-1 or both antigens in a population of patients with EC, stratified based on the presence or absence of MSI. Any study design was allowed, including case reports. Conference abstracts were excluded.

### Study Selection and Data Extraction

Titles and/or abstracts of studies retrieved using the electronic search strategy described above were screened independently by two review authors to identify studies that potentially met the inclusion criteria. Any disagreement over the eligibility of a study was resolved through discussion with a third external collaborator. Then, the full text of potentially eligible studies was retrieved and independently assessed for final eligibility. We extracted data on study characteristics (design and time of the study), study population (number and patient characteristics), methods of assessment of MSI/MSS, PD-L1/PD-1 expression and data related to the prognostic significance of PD-1 and PD-L1. A manual search of the reference lists of included studies was also performed to avoid missing relevant data.

## 3. Results

**Figure 2:**
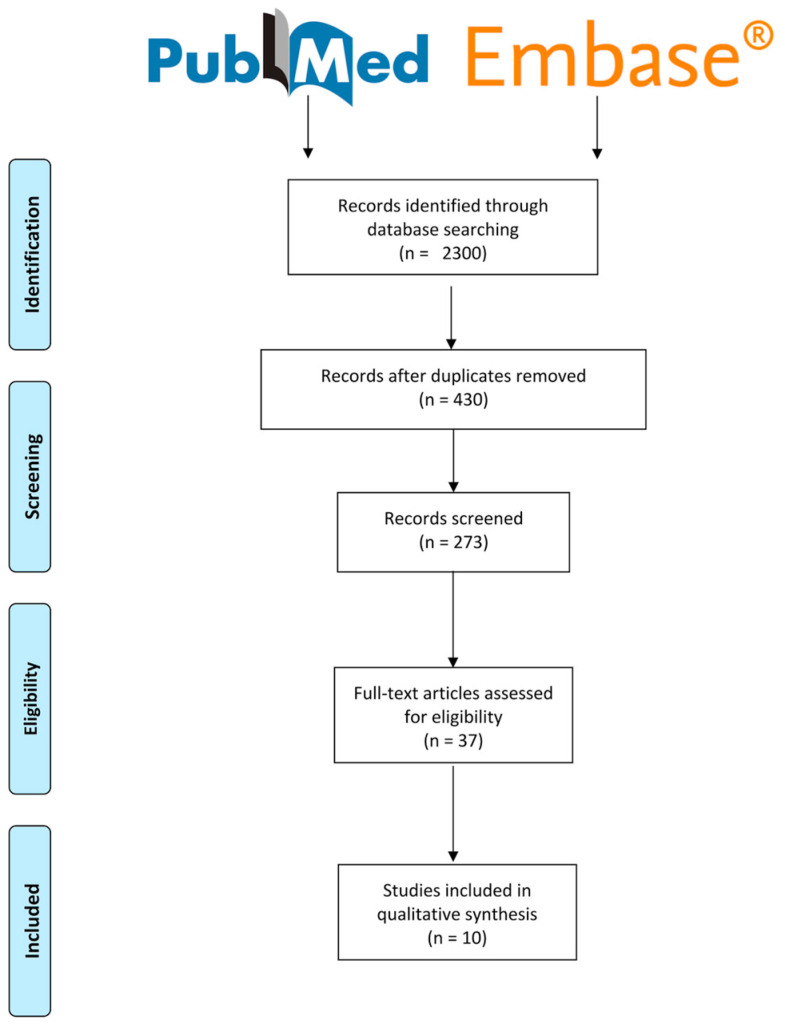
Flowchart of the systematic selection of papers.

The flowchart of the screening process is shown in Figure 2**.** A total of 2300 records were initially identified. After culling 1745 duplicates, a meticulous examination of 430 manuscripts was conducted, leading to the exclusion of several studies based on their abstracts. Full texts were acquired for 66 out of the entire 157 records. Ultimately, following a rigorous screening process, 37 full-text articles were deemed eligible for inclusion in the systematic review, all of which were composed in English. The majority of manuscripts were excluded during the screening due to disparities in study objectives (n = 16), publication types such as editorials or reviews (n = 4), and insufficient data (n = 7). A comprehensive examination identified ten studies assessing the prevalence of PD-L1 expression in patients with EC, taking into account both the populations with MSI and MSS. In particular, nine studies were retrospective, while one study was a Phase IB/II trial. Additionally, in three out of ten studies, the prevalence of PD-1 expression in patients with EC was investigated, considering both populations with MSI and MSS.

### 3.1. MSI/MSS

In a comprehensive exploration of ECs, several studies have shed light on the relationships between MSI, MMR proteins, and the expression of PD-L1 and PD-1 (Table 1). Ono et al. [17] conducted a retrospective study focusing on 17 dedifferentiated ECs from 2007 to 2017. They examined MSI through immunohistochemical (IHC) analysis of MMR proteins (MLH1, PMS2, MSH2, and MSH6) in both well-differentiated and undifferentiated components. Cases identified as MSI underwent genetic testing for dMMR/MSI-H via DNA extraction. Their findings revealed deficiencies in MMR proteins with distinct patterns between well-differentiated and undifferentiated components. Bregar et al. [18] extended the investigation to a broader spectrum, analyzing 70 uterine tumors, including high-grade endometrioid tumors, low-grade endometrioid tumors, carcinosarcomas, and uterine serous carcinomas. Among the 40 low-grade ECs studied, mutations in the four MMR proteins were assessed using IHC, uncovering dMMR/MSI-H in 33% of cases. Vanderwalde et al. [19] examined 879 cases of EC, conducting MSI analysis through a 592-gene next-generation sequencing (NGS) panel.

In a collaborative effort within the TransPORTEC consortium, Eggink et al. [20] analyzed 116 patients with EC, categorizing them based on genetic profiles. The microsatellite status of each tumor was determined using the Promega system, providing insights into the prevalence of polymerase epsilon (POLE) mutations, dMMR/MSI-H, p53 mutations, and cases with NSMP. The percentage of tumors with dMMR/MSI-H was 16% (19/116). Yamashita et al. [21] expanded the scope by evaluating a cohort of 149 individuals diagnosed with EC. Using IHC, they assessed the expression of MMR proteins, TILs (CD8+), and PD-1/PD-L1, finding that 28% of analyzed carcinomas exhibited dMMR/MSI-H. Zong et al. [22] conducted a comprehensive analysis of 833 ECs, classifying them based on molecular profiles using IHC and direct sequencing of the POLE exonuclease domain. Their study delineated subgroups, including POLE mutants, dMMR/MSI-H cases, p53 mutations, and NSMP classifications. Among these, dMMR/MSI-H tumors represented 25.7% (214/833). Rowe et al. [23], in a retrospective analysis, explored the correlation between MSI, Wnt/β-catenin pathway activation, and PD-L1 expression, considering Lynch-syndrome-associated carcinomas (23 cases), MLH1 promoter hypermethylated carcinomas (20 cases), and pMMR/MSS carcinomas. Engerud et al. [24] evaluated PD-L1 and PD-1 expression in both primary tumors and metastases. Their study included biopsies from 689 to 737 patients, which were analyzed via IHC for PD-L1 and PD-1 expression, respectively. PD-L1 was assessed in 275 corresponding metastases from 68 patients, and PD-1 expression was evaluated in 273 corresponding metastases from 74 patients. MSI was assessed using the IHC expression of PMS2 and MSH6, revealing that 9.7% of samples tested for PD-L1 and 8.7% of samples tested for PD-1 were positive. Sloan et al. [25] studied PD-L1 expression in dMMR/MSI-H ECs, including cases associated with Lynch syndrome and sporadic MLH1 hypermethylation (MLH1hm), comparing them with pMMR/MSS tumors. IHC for PD-L1/CD274 was conducted on 38 dMMR/MSI-H and 29 pMMR/MSS ECs. The KEYNOTE-146/Study 111 [26] was a multinational, open-label, single-arm study that enrolled 108 previously treated EC patients. Central testing for MSI was performed using an MSI Analysis System, while MMR status was centrally determined using a Ventana MMR Immunohistochemical Assay. Among the 108 patients, 11 (10%) exhibited dMMR/MSI-H.

**Table 1:** Studies meeting final inclusion criteria in the systematic review and their main characteristics.

Author, Year	Number of Patients	MSI	MSS	PD-L1 Positivity		PD-1 Positivity
Eggink et al., 2016 [20]	116	19 (16.4%)	97 (83.6%)	**MSI:** 13 (68.4%) **MSS:** 60 (61.8%)		**MSI:** 13 (68.4%) **MSS:** 43 (44.3%)
Sloan et al., 2017 [25]	67	38 (56.7%)	29 (43.3%)	**MSI:** 20 (52.6%) **MSS:** 3 (10.3%)		-
Bregar et al., 2017 [18]	40 *	13 (33%)	27 (67%)	*Cut-off 1%*	**MSI:** 8 (62%) **MSS:** 6 (22%)	-
				*Cut-off 5%*	**MSI:** 6 (46%) **MSS:** 2 (7%)	
Yamashita et al., 2017 [21]	149	42 (28.2%)	107 (71.81%)	**MSI:** 20 (47.6%) **MSS:** 27 (25.2%)		**MSI:** 12 (28.5%) **MSS:** 8 (7.4%)
Vanderwalde et al., 2018 [19]	879	155 (17.6%)	724 (82.4%)	**MSI:** 24 (2.7%) **MSS:** 118 (16.3%)		-
Ono et al., 2019 [17]	17	11 (64.7%)	6 (35.3%)	**MSI:** 8 (64.7%) **MSS:** 3 (35.3%)		-
Engerud et al., 2020 [24]	737 (PD-1)	64	400	-		**MSI:** 47 (73.0%) **MSS:** 241 (60.0%)
	689 (PDL-1)	60	373	**MSI:** 30 (50.0%) **MSS:** 217 (58.0%)		-
Makker et al., 2020 [26]	108	11 (13%)	94 (87%)	**MSI:** 7 (63.6%) **MSS:** 46 (48.9%)		-
Rowe et al., 2020 [23]	62	43 (69.3%): - 23 dMMR Lynch syndrome associated) - 20 (MLH1 methylated)	19 (30.6%)	*Tumoral PD-L1*	**MSI:** 26 (*60.5%*) **MSS:** 1 (*5.3%*)	-
				*Immune PD-L1*	**MSI:** 43 (100%) **MSS:** 13 (*68.4%*)	
Zong et al., 2021 [22]	833 (587 subcategorized based on molecular classification)	163 (27.2%)	433 (73.8%) - 49: POLE mut - 254: NSMP - 130: p53 abn	**MSI** - Immune cells: 129 - Tumor cell: 43 - Combined positive score: 149 **MSS** - Immune cells: 178 - Tumor cell: 70 - Combined positive score: 220		-

Values are expressed as n (%), unless otherwise specified. * Low-grade endometrioid subtypes. MSI: Microsatellite Instability; MSS: Microsatellite Stable; PD-1: Programmed Death-1; PD-L1: Programmed Death-Ligand 1; POLE mut: Polymerase epsilon-mutated; dMMR: Mismatch Repair Deficient; p53 abn: Abnormal p53 expression; NSMP: No Specific Molecular Profile.

### 3.2. PD-L1 Assessment

In the study by Ono et al. [17], PD-L1 assessment through IHC was considered positive when staining was observed in more than 5% of tumor cells. Yamashita et al.’s study [21] expanded the criteria for positivity by including staining in ≥5% of tumor cells, accounting for both membranous and cytoplasmic staining. In contrast, Bregar et al.’s investigation [18], also utilizing IHC for PD-L1 evaluation, defined positivity as staining exceeding 1% of tumor cells. PD-L1 expression was observed in 6 out of 27 low-grade endometrioid MSS tumors and 8 out of 13 low-grade endometrioid dMMR/MSI-H tumors. However, when applying a threshold of >5% for positivity, PD-L1 was positive in only 2 out of 27 pMMR/MSS cases and in 6 out of 13 dMMR/MSI-H cases.

Eggink et al. [20] and Vanderwalde et al. [19] employed IHC for PD-L1 assessment, deeming it positive when detectable in ≥1% of tumor cells. Zong et al. [22] and Makker et al. [26] utilized the Combined Positive Score (CPS) as the scoring algorithm, defined as the total number of PD-L1-positive cells (tumor cells, lymphocytes, and macrophages) divided by the total number of viable tumor cells within the evaluated area, multiplied by 100. CPS was considered positive when staining was detected in ≥1% of tumor and/or immune cells.

Sloan et al. [25] evaluated PD-L1 IHC staining, encompassing both the tumor and peritumoral immune compartments. Positive tumor staining was determined by definitive membranous positivity, stratified according to the percentage of cells stained with the antibody. Finally, Engerud et al. [24] used a staining index for PD-L1 assessment, calculated as the product of staining intensity and the area of positive tumor cells expressed as a percentage.

### 3.3. PD-1 Assessment

Among the studies included in this analysis, the expression of PD-1 was assessed in three studies using IHC. Eggink et al. [20] quantified PD-1-positive cell numbers per core. They estimated the percentage of tumor and stromal surface area within each core and used these data to project cell counts across the entire surface area. Using a median value of 14 PD-1-positive cells per core for the entire patient cohort, tumors were classified as highly infiltrated with PD-1. In the study by Yamashita et al. [21], PD-1 expression was considered positive if ≥5% of tumor cells exhibited staining, including both membranous and cytoplasmic staining. Conversely, in Engerud et al.’s study [24], PD-1 expression was classified as positive when 5% of stromal cells showed staining.

## 4. Systematic Review

The included studies, published between 2016 and 2021, analyzed a total of 3486 patients. Among the 10 studies, molecular classification was performed in 2, tumor grading stratification in 6, and tumor histology was specified in all.

### 4.1. MSI/MSS and PD-L1/PD-1

A PD-L1 expression analysis was conducted in a subset of 2457 patients, comprising 555 patients (23%) with MSI and 1902 (77%) with microsatellite stability. PD-L1 expression was detected in 272 of the 555 dMMR/MSI-H cases, corresponding to 49% (95% CI: 45–53%) of this subgroup and 11% of the total population. In pMMR/MSS cases, PD-L1 expression was found in 637 of 1902 cases, representing 33.5% (95% CI: 31.5–35.5%) of this subset and 26% of the total population. Overall, PD-L1 expression was observed in 909 patients (37%), with a two-tailed *p* value of < 0.0001.

PD-1 expression was analyzed in 729 patients, including 125 (17%) with MSI and 604 (83%) with microsatellite stability. PD-1 expression was detected in 72 dMMR/MSI-H cases, accounting for 58% (95% CI: 50–66%) of this subgroup and 10% of the total population. Among pMMR/MSS cases, PD-1 expression was observed in 291 patients, representing 48% (95% CI: 44–52%) of this subset and 40% of the total population. Across the entire cohort, PD-1 expression was detected in 364 patients (50%). The two-tailed *p* value was 0.0625.

### 4.2. POLEmut/NMSP/p53mut and PD-L1

Within the pMMR/MSS population, molecular subtyping was conducted in only two studies [20,22]. PD-L1 expression was observed in 49 of 64 cases (76.56%) within the POLE-mutated subgroup, 79 of 248 cases (31.85%) in the NMSP subgroup, and 67 of 172 cases (39%) in the p53-mutated subgroup.

### 4.3. Tumor Grading (G1/G2-G3), Histotypes, and Metastatic Lesions

Among patients classified by tumor grade, 804 had low-grade (G1/G2) tumors, and 954 had high-grade (G3) tumors. MSI was present in 47% of both low-grade (379/804) and high-grade (449/954) cases. PD-L1 expression was observed in 316 of 643 low-grade tumors (49%) and in 315 of 738 high-grade tumors (42%).

By histotype, PD-L1 expression was noted in 50.7% (604/1191) of endometrioid carcinomas, 56% (40/71) of clear cell carcinomas, 73% (102/178) of serous carcinomas, 37% (31/84) of carcinosarcomas, and 72% (21/29) of dedifferentiated carcinomas.

One study examined PD-L1 expression in metastatic lesions, revealing significant discordance between primary tumors and metastatic sites, as well as variability among multiple metastases from the same patient [24].

### 4.4. Prognostic Value

Zong et al. [22] found no significant association between PD-L1 expression in tumor cells and overall survival (OS). However, PD-L1 expression was linked to favorable outcomes in FIGO stage II–IV, non-endometrioid endometrial cancer (NEEC), and high-risk subgroups. Yamashita et al. [21] reported no significant differences in progression-free survival (PFS) or OS between dMMR/MSI-H and pMMR/MSS groups but observed prolonged PFS in PD-L1-positive cases, with no OS benefit.

Engerud et al. [24] found no significant impact of PD-L1 or PD-1 on disease-specific survival (DSS), with PD-L1 lacking prognostic value in both endometrioid and non-endometrioid histologies. However, PD-1 positivity was associated with better DSS in endometrioid cancers (5-year DSS: 96% vs. 89%, *p* = 0.02) but NEEC (5-year DSS: 60% vs. 41%, *p* = 0.23).

In dMMR/MSI-H tumors, while PD-L1 and PD-1 were linked to favorable clinical features, this did not translate into improved survival outcomes.

## 5. Discussion

This study represents the first systematic analysis in the literature to evaluate PD-L1 and PD-1 expression rates in EC by incorporating data from all available studies that stratified the expression of these two antigens based on microsatellite status. Our findings indicate that PD-L1 is expressed in 49% of dMMR/MSI-H cases (11% of the total population) and 33.5% of pMMR/MSS cases (26% of the total population). Similarly, PD-1 expression was observed in 58% of dMMR/MSI-H cases (10% of the total) and 48% of pMMR/MSS cases (40% of the total). Across the entire cohort, 37% of patients expressed PD-L1, while 50% expressed PD-1.

These results are particularly significant in the context of the increasing use of ICIs. In EC, however, comprehensive evaluation of PD-L1 and PD-1 status has been limited. Current clinical decision-making primarily relies on mismatch repair (MMR) status, which determines eligibility for ICIs.

Since the FDA’s first approval of an ICI in March 2011, these therapies have revolutionized the treatment landscape for numerous malignancies. For EC, the FDA approved dostarlimab in April 2021 for adult patients with recurrent or advanced dMMR/MSI-H EC, based on the Phase II GARNET trial [27]. In March 2022, pembrolizumab received approval for use as a monotherapy in advanced dMMR/MSI-H EC following promising results from the Phase II KEYNOTE-158 trial [28]. Both therapies target PD-1. While both GARNET [27] and KEYNOTE-158 [28] trials supported the efficacy of PD-1 inhibitors, they did not evaluate the role of biomarkers such as PD-L1 or PD-1. For the pMMR/MSS population, subsequent trials like KEYNOTE-146 [26] and KEYNOTE-775 [14] explored combination therapies, pairing pembrolizumab with the antiangiogenic agent lenvatinib. The Phase II KEYNOTE-146 trial demonstrated significant efficacy in both dMMR/MSI-H and pMMR/MSS patients, and the Phase III KEYNOTE-775 trial [14] showed that this combination improved PFS in pMMR/MSS patients (6.6 months vs. 3.8 months compared to doxorubicin or paclitaxel).

However, these advancements come with challenges. Severe adverse events (Common Terminology Criteria for Adverse Events grade ≥3) occurred in 88.9% of patients treated with pembrolizumab plus lenvatinib, compared to 72.7% in the chemotherapy group.

More recently, trials like NRG-GY018 [29] and RUBY [30] have expanded the scope of immunotherapy by incorporating ICIs into frontline treatment regimens, regardless of microsatellite status. In the NRG-GY018 trial [29], the pMMR/MSS population exhibited a 46% reduction in the risk of progression or death in the pembrolizumab group compared to placebo. Similarly, the RUBY trial [30] demonstrated a slight improvement in OS at 24 months in pMMR/MSS patients treated with dostarlimab (67.7% vs. 55.1% in the placebo group; HR: 0.73), whereas for dMMR/MSI-H patients, there was a substantial PFS advantage with dostarlimab (57.0% vs. 10.2%; HR: 0.31) [31].

While these findings highlight significant PFS and OS improvements in dMMR/MSI-H patients, the partial benefits observed in pMMR/MSS populations underscore the need to identify pMMR/MSS subgroups that may benefit most from ICIs.

Our analysis sheds light on PD-L1 and PD-1 expression across dMMR/MSI-H and pMMR/MSS populations. Notably, PD-L1 and PD-1 expression were observed in subsets of pMMR/MSS patients, traditionally not considered immunotherapy candidates. In our study, 37% of the total population expressed PD-L1, and 50% expressed PD-1. These findings suggest that evaluating antigenic expression beyond MMR status could expand the pool of patients eligible for immunotherapy, potentially improving survival outcomes.

The higher expression of PD-1 compared to its ligand may explain why phase III trials in pMMR/MSS populations report higher response rates with PD-1 inhibitors (e.g., pembrolizumab, dostarlimab) [29,30] compared to PD-L1 inhibitors (e.g., avelumab, durvalumab, atezolizumab) [32,33,34]. This discrepancy suggests that PD-1 activation might also be mediated by alternative ligands, such as PD-L2 [35]. Further translational research is needed to explore these mechanisms.

Interestingly, high PD-L1 expression was observed in aggressive histologic subtypes, including clear cell, serous, and dedifferentiated carcinomas, with rates exceeding 70% in some subtypes. This underscores the potential role of ICIs in high-risk patients, a topic being explored in ongoing clinical trials [36].

In the POLE-mutated subgroup, PD-L1 expression was similarly elevated, consistent with evidence linking POLE mutations to genomic instability and immunogenic phenotypes. These tumors are associated with increased TILs and immune biomarker expression, including PD-L1 [37], suggesting that POLE status may serve as a novel predictive biomarker for ICI responsiveness.

Regarding its prognostic value, PD-L1 expression is widely recognized as an adverse prognostic factor in several malignancies, such as gastric cancer, hepatocellular carcinoma, renal cell carcinoma, esophageal cancer, pancreatic cancer, and bladder cancer [38]. However, its prognostic relevance in EC remains ambiguous. Previous evidence suggests that PD-L1 overexpression has a non-significant impact on overall survival (OS) [39]. Nonetheless, the data presented in this review remain partial, inconclusive, and inconsistent. Although the expression of PD-1 and PD-L1 appears to correlate with improved progression-free survival (PFS) in certain subgroups, the findings lack reproducibility across studies and display conflicting patterns. Moreover, the studies often fail to consider tumor staging or treatment regimens, further complicating data interpretation. A more comprehensive and stratified analysis, incorporating treatment protocols and patient demographics, is necessary to better elucidate its prognostic significance.

### Limitations

The main limitations of our pooled analysis stem from several methodological challenges, particularly the variation in population sizes and the heterogeneity of study designs. Among the studies included in the final systematic review, those that did not provide a numerical estimate of PD-L1/PD-1-positive ECs relative to the total population or lacked stratification based on MSI were excluded. Additionally, studies for which the full text was inaccessible or where the data were uninterpretable were also omitted. Only three studies evaluated PD-1 expression, each involving a small sample size, further limiting the robustness of the findings.

Another significant limitation of this review is the lack of prospective studies and randomized controlled trials, which introduces the potential for publication and selection bias. Moreover, nearly all included studies demonstrated a high risk of participant selection bias due to the use of unrepresentative patient cohorts. Variability in the methods used to assess PD-L1/PD-1 expression across studies also introduces potential outcome bias. For instance, while both IHC and flow cytometry are employed to detect PD-L1 expression in clinical settings, traditional IHC techniques face challenges in accuracy and reliability due to issues such as cytoplasmic protein staining interfering with the evaluation of cell membrane proteins. Conversely, flow cytometry, while highly specific, is limited to fresh tissue samples, which restricts its applicability in broader clinical contexts.

In the current review, PD-L1 expression is determined in most studies using the Tumor Proportion Score (TPS), which represents the percentage of viable tumor cells exhibiting partial or complete membrane staining at any intensity. In the current review, the threshold for defining PD-L1 positivity on tumor cells varies among different studies ranging from 1% to 50%. However, within the EC landscape, a comparative evaluation of these thresholds is not feasible due to the lack of studies correlating TPS with objective response rates. Occasionally, classifying PD-L1 expression in tumor tissue as either positive or negative based solely on TPS may lack precision. The use of the PD-L1 Combined Positive Score (CPS) provides a more comprehensive approach by accounting for PD-L1 expression in both tumor and non-tumor cells. For this reason, CPS is considered the reference method for assessing PD-L1 expression in other oncological contexts and could potentially offer greater relevance in EC studies [40]. Recently, non-invasive methodologies employing radionuclides to quantify PD-L1 expression have been described in the literature. The integration of such approaches into clinical practice could represent a novel frontier in the study of ICIs and their interactions with other therapeutic agents [41,42].

## 6. Implications for Practice and Future Research

Currently, international guidelines do not recommend the routine assessment of PD-L1 and PD-1 in the molecular screening of EC. However, growing scientific evidence suggests the potential role of ICIs as frontline therapy, particularly when combined with traditional chemotherapy, regardless of microsatellite status.

In the recurrent setting, the combination of pembrolizumab and lenvatinib is currently the preferred option for the pMRR/MSS subset, despite the significant morbidity associated with this pharmacological regimen. This review highlights that PD-L1 is expressed in up to one-third of cases, while PD-1 is expressed in up to 50% of the pMMR/MSS population. Consequently, assessing PD-L1 expression in recurrent pMMR/MSS cases could help identify patients who might benefit from monotherapy with ICIs.

Furthermore, the recently published DUO-E/GOG-3041/ENGOT-EN10 trial evaluated the addition of the PARP inhibitor olaparib to durvalumab during maintenance therapy. The trial revealed that pMMR/MSS patients with positive PD-L1 expression particularly benefited from the synergistic effect of these two agents, showing significantly improved PFS outcomes [43].

These findings underscore the potential utility of PD-L1 assessment in optimizing personalized treatment strategies for advanced EC.

## 7. Conclusions

This study provides valuable insights into the expression of PD-L1 and PD-1 in EC, emphasizing their potential relevance in expanding therapeutic options, particularly in the pMMR/MSS population, suggesting a broader patient group that could benefit from ICIs beyond the current focus on MMR status. However, the limited data currently available highlight the need for further research, particularly studies that correlate PD-L1/PD-1 expression in primary and metastatic lesions. Future investigations with well-defined control groups are essential to fully understand the therapeutic impact of PD-L1 and PD-1 expression and to better inform personalized treatment strategies for patients with EC.

## Data Availability

Upon request.
